# An Interstitial Deletion at 7q33-36.1 in a Patient with Intellectual Disability, Significant Language Delay, and Severe Microcephaly

**DOI:** 10.1155/2016/6046351

**Published:** 2016-12-08

**Authors:** Trupti Kale, Melissa Philip

**Affiliations:** The Wright Center for Graduate Medical Education, 501 Madison Avenue, Scranton, PA 18510, USA

## Abstract

Interstitial deletions of the distal 7q region are considered a rare entity. In this report, we describe a seven-year-old male with a heterozygous interstitial deletion at 7q33-36.1 with characteristic dysmorphic facial features, intellectual disability, severe microcephaly, and significant language delay. The primary focus of our report is to compare our case with the few others in the literature describing interstitial deletions at the long arm of chromosome 7. Based on the various breakpoints in prior studies, a number of phenotypic variations have been identified that are unique to each of the reports. However, there are also a number of similarities among these cases as well. We hope to provide a concise review of the literature and genes involved within our deletion sequence in the hope that it will contribute to creating a phenotypic profile for this patient population.

## 1. Introduction

Terminal deletions at the long arm of chromosome 7 have been described more frequently in the medical literature [1, 2] compared to interstitial deletions at the distal 7q region. Patients with terminal deletions often have severe phenotypic features including sacral agenesis and holoprosencephaly [2, 3], whereas, the above mentioned interstitial deletions have characteristic dysmorphic facial features, intellectual disability, and language impairment which will be further described in this report. Among distal 7q interstitial deletions, the involvement of the 7q36.1 and 7q36.2 has only been evaluated by cytogenetic techniques in a few reports [4–7]. A specific distal 7q interstitial deletion syndrome has not been proposed because of the rarity of these deletions and the various breakpoints described in prior studies. The aim of this case report is to further understand the phenotypic features associated with interstitial deletions at the long arm of chromosome 7 and offer a basis for the development of a possible syndromic profile for these deletions in the future.

## 2. Case Presentation

The patient described in our study was born at term via spontaneous vaginal delivery to a 16-year-old, G1P0 mother and a 17-year-old father. Birth weight was eight pounds and there were no maternal complications during pregnancy or at birth. Neonatal course was only complicated by jaundice requiring phototherapy. At birth, the patient did not have any dysmorphic features, except for an umbilical hernia and microcephaly. Family history is significant for a paternal aunt with developmental delay, requiring special education services. There is no history of intellectual or learning disabilities in any first degree relatives. The patient began to show signs of developmental delay early in his Denver Developmental Assessments. At 18 months, he was still unable to walk or even crawl or cruise and was only able to say “mama.” Patient failed his 18-month M-CHAT assessment. He was referred to a neurologist and early intervention developmental therapy for not meeting developmental milestones in regard to gross motor skills and lack of speech development.

By 21-months, patient began to walk with one-hand held. He had no history of seizures and a normal extended EEG study. An MRI of the brain was performed and showed cavum septum pellucidum and verge as well as small optic nerves and chiasma. Although he did not meet diagnostic criteria for septooptic dysplasia, the findings may likely represent a congenital syndrome within the broad spectrum of septooptic dysplasia. At 24 months, he had an ophthalmologic exam which showed left eye esotropia and amblyopia and anomalous optic nerves with the left being smaller than the right. Audiometry exams did not show evidence of hearing impairment. Due to global developmental delay, he had a chromosomal microarray analysis at 28 months. Parental genotyping could not be obtained at that time due to insurance coverage.

Dysmorphic features in this individual include severe microcephaly (OFC less than 1st percentile), short stature, right frontal cowlick, abnormally shaped pinnae, thick overfolded helices, low nasal columella, bulbous nose, smooth, broad philtrum, thin upper lip, mild retrognathia, long slender fingers, and mild genu valgum. Patient was also regularly being evaluated by a neurodevelopmental pediatrician. By 5 years of age, neurobehavioral observation showed appropriate social reciprocity for developmental age with referential gaze but difficulty maintaining attention. Clinical Linguistic and Auditory Milestone Scale (CLAMS) at age 5 revealed receptive language age equivalent to 16 months, expressive language age equivalent to 12 months, and speech age equivalent to 10 months.

Throughout the course of his well visits, he never had a regression in his developmental milestones. At age 7, patient now shows improvement in using gestures such as pointing to communicate, but his overall vocabulary is limited to about 10 words, without any two-word combinations. Gross motor skills have evolved. Patient is able to kick and throw a ball but has difficulty with uneven surfaces and is unable to pedal a bike. He is still not toilet-trained. Currently he is in special education classes. He also receives occupational, speech, and visual therapy.

We have referred our patient for Augmentative and Alternative Communication (AAC) evaluation which utilizes a multimodal approach for individuals with severe, expressive communication disorders. AAC employs aided and unaided forms of communication. Unaided communication consists of gestures, facial expressions, and learning American Sign Language. Aided communication uses more innovative methods via technological advancements with electronic communication devices or more traditional communication boards with visual graphic symbols. We have also encouraged his parents to learn sign language to enhance his nonverbal communication at home. Rather than impede further language acquisition, sign language is considered to accelerate speech development.

## 3. Cytogenetics

The molecular technique used for cytogenetic analysis was Array Comparative Genomic Hybridization (aCGH) analysis with confirmatory G-banded chromosome analysis. A 13.8 Mb interstitial deletion of the long arm of chromosome 7 involving bands q33 to q36.1 was identified. It revealed arr 7q33-q36.1 deletion (chr7: 134,358,978–148,135,425; hg18). aCGH analysis was performed utilizing a custom oligonucleotide array, known as EmArray Cyto 60K (Emory Genetics Laboratory, Atlanta, GA) that was designed to detect copy number imbalances of specific chromosomal regions across the genome. The array was built using a platform from Agilent Technologies and contains about 60,000 oligonucleotides that represent coding and noncoding sequences (UCSC hg18; NCBI Build 36.1, March 2006) that are designed to detect gains and losses across the genome by combining targeted and genome-wide array analyses. The genome-wide coverage has an average probe spacing of about 75 kb. Specimen used was whole blood. Successful hybridization was indicated by ratio values and sex chromosome positive controls that were within acceptable ranges. Fragile X syndrome molecular analysis was also performed and detected a normal 24 CGG repeat within the FMR1 gene.

## 4. Discussion

In reviewing the literature, we have not discovered any other patients with the exact breakpoints described in our report. However, there are individuals with similar overlapping interstitial deletions of the distal 7q region.  Table 1 compares the clinical phenotype of our patient with these individuals. Either intellectual disability or a form of autism spectrum disorder appears to be a common finding in all the patients. They all exhibit some form of speech delay and most of them have attention deficits and history of a seizure disorder. Deep-set eyes, bulbous nasal tip, and hypertelorism are the most common craniofacial dysmorphisms. Many of these patients also display some type of ear abnormality, whether it is an abnormal pinnae, low set ears, or preauricular pits. Short stature and various ophthalmologic abnormalities are also noted. Our patient has left esotropia, amblyopia, and variation in the size of the optic nerves. Dilzell et al. report a patient with hyperopia and bilateral astigmatism [8]. More deleterious ocular abnormalities include optic nerve atrophy [7] and iris-retina-nervus opticus coloboma [4].

Less common craniofacial dysmorphisms include cleft lip and palate, microcephaly, hypermetropia, epicanthal folds, micrognathia, and retrognathia. Only one other patient, other than Rush et al.'s patient [6], had cleft lip and palate [11]. Microcephaly was reported in our patient and by Bisgaard et al. [4]. Microcephaly is associated with a genetic etiology in 15.5 to 53.3% of cases [12]. Severe microcephaly is more likely to be associated with imaging abnormalities, as seen in our patient. His brain MRI revealed cavum septum pellucidum and verge which is considered a common anatomical variant. Individuals with microcephaly are at increased risk for the following disorders: epilepsy (about 40%), cerebral palsy (about 20%), mental retardation (about 50%), and ophthalmologic disorders (about 20%–50%) [12].

Interestingly, the patient reported in this study exhibits severe microcephaly but has no history of seizure activity to date and a normal EEG in the past. However, the patients reported by Bisgaard et al. have microcephaly and history of seizures [4]; both twins had febrile seizures and one developed tonic-clonic seizures at a later date. There is also an association between epilepsy and dysrhythmia within the interstitial deletion sequence.* KCNH2*, a gene located at 7q36.1, encoding voltage gated potassium channels has been linked to Long QT syndrome type 2 as well as epilepsy [13]. Both patients in the literature who have Long QT syndrome also have a history of seizure activity [4, 5]. We obtained an electrocardiogram (ECG) for our patient due to concern for QT prolongation associated with the* KCNH2* gene and in order to avoid medications which can worsen the condition. Fortunately, it only showed left atrial enlargement.

Both intellectual disability and attention deficits appear to be significant in our patient. He was diagnosed with intellectual disability based on Developmental Profile-3 testing where he consistently fell below two standard deviations in assessment of adaptive behavior, social-emotional functioning, cognitive development, and communication skills. The detailed report is presented in Table 2. At age 5, he was evaluated based on the Conners Early Childhood Global Index (CGI) scale. The CGI raw and* T*-scores are listed in Table 3. His* T*-scores were markedly atypical in the 98th percentile, considered “very elevated score” based on the grading scale. Characteristics of high scorers include being easily distracted, restless, fidgety, and/or impulsive, having difficulty completing assignments, distracting others, and having increased fluctuations in mood and emotions.

Speech delay is another common finding among all the distal 7q interstitial deletions reported. Patients have more difficulty with expressive rather than receptive language. Despite early intervention and speech therapy, our patient's communication skills continue to be very poor. He also exhibits poor social communication, initially concerned for possible autism spectrum disorder. However, he does not have any stereotypic behaviors, echolalia, or other restricted, repetitive patterns of behavior or problems with nonverbal communication. Patient has also been evaluated at an Autism and Developmental Medicine Institute. Based on their assessment, his behavior is more closely linked to his intellectual disability. One gene within the deleted region associated with speech delay and autism spectrum disorders is* CNTNAP2* [14].* CNTNAP2* (contactin-associated protein-like 2) is located on 7q35-36. This gene expresses a neuronal transmembrane protein of the neurexin superfamily which is mainly involved in neural-glia interactions [15]. Petrin et al. also report that this gene is involved in higher cortical regions of the brain significant for language delay [16]. They report a Brazilian stuttering case where the sites of* FOXP2* binding were also affected by deletion of* CNTNAP2*. Both genes have a common neurogenic pathway that is associated with language and speech development.* CNTNAP2* may be implicated in our patient's severe speech delay.

Often, distal 7q deletions are de novo. Nevertheless, we have recommended genetic counseling to parents for further testing and expectations for future pregnancies. There is also additional testing necessary to monitor the patient's phenotypic profile over time. He needs a renal ultrasound to evaluate renal hypoplasia. We also plan to do serial ECGs to assess any changes in his cardiac status.

## 5. Conclusion

The aim of this study is to compare our case with prior reports to suggest a phenotypic profile associated with distal 7q interstitial deletions. Based on our assessment of the current literature, we predict that the majority of individuals with these deletions will present with intellectual disability, language and growth delay, attention deficits, and epilepsy. Craniofacial features will likely include deep-set eyes, hypertelorism, bulbous nose, ear deformities, and ophthalmologic abnormalities. This will provide a framework to identify patients with similar interstitial deletion sequences of the distal 7q region and aid in early interventions for intellectual disability and speech delay.

## Figures and Tables

**Table 1:** Clinical features in patients with interstitial deletions overlapping the 7q33-36.1 region.

Characteristic traits	Present study	Rush et al., 2013 [6]	Sehested et al., 2010 [7]	Sehested et al., 2010 [7]	Dilzell et al., 2015 [8]	Bisgaard et al., 2006 [4]	Fagan et al., 1994 [9]	Rossi et al., 2008 [10]	Caselli et al., 2008 [5]
Chromosomal deletion	7q33-36.1	7q34-36.1	7q34-36.2	7q34-36.2	7q33-35	7q34-36.2	7q35	7q33-35	7q36.1-36.2
Gender	Male	Female	Male	Female	Female	Females (twins)	Female	Female	Female
*Neurodevelopmental features*									
Developmental delay/	+	+		+	+	+	+		+
intellectual disability									
Autism spectrum disorder			+					+	
Attention deficit	+	+		+		+			+
Language delay	+	+	+	+	+	+	+	+	+
Abnormal brain MRI	+				+				
Seizures	−	+	+	+	+	+		+	+
Insomnia	+				+			+	
*Craniofacial features*									
Microcephaly	+	−	−	−		+			
Deep-set eyes	−	+	+	+		+		+	+
Hypermetropia	−	−	−	+		+			
Hypertelorism	−	−	+	+	+	−		+	
Epicanthal folds	−	−					+		
Broad/depressed nasal bridge		+						+	+
Bulbous nose	+	+	+	+	+	+	+	+	+
Abnormal philtrum	+	−			+			+	
Thin upper lip	+	−			+	−			
Cleft lip/palate	−	+	−	−	−	−	−	−	−
Micrognathia	−	−					+		
Retrognathia	+	−							
Low set ears	−	−		+			+		
Preauricular pits	−	−			+			+	
Abnormal pinnae	+	+							
*Other*									
Hearing impairment	−	+	−	−	+	+			−
Short stature	+	+	+	+		+		+	−
Long slender fingers	+							+	
Ophthalmologic abnormality	+			+	+	+	+		
Long QT syndrome	−	−	−	−		+			+
Renal hypoplasia			−	−		+			+

**Table 2:** Developmental profile: 3 at age 5.

	Age equivalent (months)	Standard score
Adaptive behavior	18	<50
Social-emotional	18	<50
Cognitive	18	<50
Communication	12	<50

Standard scores have a mean of 100 and a standard deviation of 15.

**Table 3:** Connors Early Childhood Global Index scores.

	Raw score	*T*-score
Restless-impulsive	16	79
Emotional lability	8	87
Total	24	82

*T*-scores have a mean of 50 and a standard deviation of 10.
